# Nasopharyngeal tubes in pediatric anesthesia: Is the flow‐dependent pressure drop across the tube suitable for calculating oropharyngeal pressure?

**DOI:** 10.1111/pan.14194

**Published:** 2021-05-06

**Authors:** Paola Papoff, Talitha Rosini, Salvatore Oliva, Stefano Luciani, Fabio Midulla, Francesco Montecchia

**Affiliations:** ^1^ Paediatric Intensive Care Unit Department of Paediatrics Sapienza University of Rome Rome Italy; ^2^ Medical Engineering Laboratory Department of Civil Engineering and Computer Science Engineering University of Rome “Tor Vergata” Rome Italy; ^3^ Paediatric Gastroenterology and Liver Unit Department of Paediatrics Sapienza University of Rome Rome Italy; ^4^ Paediatric Emergency Care Department of Paediatrics Sapienza University of Rome Rome Italy

**Keywords:** anesthesia, nasopharyngeal tube, oropharyngeal pressure, pediatrics, pressure drop, Rohrer's equation

## Abstract

**Background:**

Nasopharyngeal tubes are useful in pediatric anesthesia for insufflating oxygen and anesthetics. During nasopharyngeal tube‐anesthesia, gas insufflation provides some positive oropharyngeal pressure that differs from the proximal airway pressure owing to the flow‐dependent pressure drop across the nasopharyngeal tube (ΔP_NPT_).

**Aims:**

This study aimed to investigate whether ΔP_NPT_ could be used for calculating oropharyngeal pressure during nasopharyngeal tube‐assisted anesthesia.

**Methods:**

In a physical model of nasopharyngeal tube‐anesthesia, using Rohrer's equation, we calculated ΔP_NPT_ for three nasopharyngeal tubes (3.5, 4.0, and 5.0 mm inner diameter) under oxygen and several sevoflurane in oxygen combinations in two ventilatory scenarios (continuous positive airway pressure and intermittent positive pressure ventilation). We then calculated oropharyngeal pressure as proximal airway pressure minus ΔP_NPT_. Calculated and measured oropharyngeal pressure couples of values were compared with the root mean square deviation to assess accuracy. We also investigated whether oropharyngeal pressure accuracy depends on the nasopharyngeal tube diameter, flow rate, gas composition, and leak size. Using ΔP_NPT_ charts, we tested whether ΔP_NPT_ calculation was feasible in clinical practice.

**Results:**

When we tested small‐diameter nasopharyngeal tubes at high‐flow or high‐peak inspiratory pressure, proximal airway pressure measurements markedly overestimated oropharyngeal pressure. Comparing measured and calculated maximum and minimum oropharyngeal pressure couples yielded root mean square deviations less than 0.5 cmH_2_O regardless of ventilatory modality, nasopharyngeal tube diameter, flow rate, gas composition, and leak size.

**Conclusion:**

During nasopharyngeal tube‐assisted anesthesia, proximal airway pressure readings on the anesthetic monitoring machine overestimate oropharyngeal pressure especially for smaller‐diameter nasopharyngeal tubes and higher flow, and to a lesser extent for large leaks. Given the importance of calculating oropharyngeal pressure in guiding nasopharyngeal tube ventilation in clinical practice, we propose an accurate calculation using Rohrer's equation method, or approximating oropharyngeal pressure from flow and pressure readings on the anesthetic machine using the ΔP_NPT_ charts.

**What is already known about the topic**

- In anesthetized spontaneously breathing children, a nasopharyngeal tube connected to a flow‐inflating bag can help deliver oxygen and anesthetics and improve airway patency by providing positive oropharyngeal pressure.
- In intubated children, the pressure gradient across endotracheal tubes is conventionally calculated to determine the distal pressure, a variable that is clinically more relevant than the proximal airway pressure because it identifies the force applied to biological tissue.

**What new information this study adds**

- In children under nasopharyngeal tube‐anesthesia, the pressure drop across the tube is suitable for calculating oropharyngeal pressure using Rohrer's equation and its coefficients (K1 and K2).
- Rohrer's coefficient K2 increases under sevoflurane anesthesia and increases more for smaller than for larger tubes.
- Assessing oropharyngeal pressure will allow anesthesiologists, who use nasopharyngeal tube‐anesthesia, to estimate the real pressure applied to the airway, a measure that unlike the proximal airway pressure reading is unaffected by small‐diameter tube size.

## 1 INTRODUCTION

In adults and children, among the many possible ways to manage the upper airway, one involves placing an endotracheal tube in the oropharynx (nasopharyngeal tube, NPT). The NPT has proved useful to deliver continuous positive airway pressure (CPAP) in premature infants^1^ and to facilitate intermittent positive pressure ventilation (IPPV) in several emergency conditions.^2^ In anesthetized spontaneously breathing adult patients, a customized nasopharyngeal airway, the nasal trumpet, was used to facilitate elective and semi‐elective fiber optic intubation.^3^ In this setting, the nasal trumpet helped to establish a patent airway and deliver positive pressure ventilation, without impeding fiber optic tracheal intubation. In pediatrics, a similar experience with the NPT comes from Holm‐Knudsen and colleagues, who used this technique to help fiber optic intubation in small children with a difficult airway (ie, Pierre Robin sequence, Treacher Collins, and similar syndromes).^4^

A procedure that can deliver oxygen, anesthetics and some positive pressure is essential during spontaneously breathing anesthesia. Preserving a balance between adequate sedation and effective ventilation is challenging for anesthesiologists caring for children. Young children have efficacious protective reflexes that require deep sedation for suppression. Children are particularly susceptible to upper airway obstruction because they have small‐diameter airways and a high incidence of tonsillar and adenoidal hypertrophy, which increases resistance to flow. Because the upper airway consists of soft tissue and during inspiration is kept patent by pharyngeal airway muscle dilation, drugs that reduce muscle activity can reduce airway patency, often at the velopharyngeal level, and, thus, increase upper airway resistance.^5^ Hence, many anesthesiologists resort to intubation even for procedures that require only minutes to complete.

To avoid unnecessary intubation, an NPT can be placed with the tip just above the larynx and positive pressure can be applied at the oropharynx level. Setting flow to a target oropharyngeal pressure or having reliable pressure readings upon increasing the flow rate is difficult during NPT‐assisted anesthesia partly owing to gas leakage through the mouth, but, also, because the resistance offered by the small NPT diameter makes the airway pressure measured at the proximal end of the tube (proximal airway pressure, Paw) higher than the pressure measured at the distal end (Pdist or oropharyngeal pressure). This pressure difference, that is, the flow‐dependent pressure drop across the NPT (ΔP_NPT_), depends on flow characteristics (rate, direction, and acceleration), gas composition and the tube diameter, length, curvature, and material.^6^, ^7^

As an alternative to the troublesome dedicated pressure catheter system, oropharyngeal pressure can be calculated from the Paw reduced by the ΔP_NPT_ (oropharyngeal pressure = Paw − ΔP_NPT_). This mathematical construct has been successfully used for calculating pressure downstream an endotracheal tube (intra‐tracheal pressure).^8^ No published study has to date attempted to calculate oropharyngeal pressure during NPT‐assisted anesthesia from the ΔP_NPT_. If this method proved effective, it would provide the basis for implementing anesthetic machines with algorithms for calculating oropharyngeal pressure during NPT ventilation. It would also allow physicians to know what pharyngeal pressure they are actually delivering during CPAP or IPPV when changing the flow rate or pressure.

In this study, we aimed to investigate whether characterizing the ΔP_NPT_ for various tube sizes under various gas compositions would enable us to calculate oropharyngeal pressure from Paw and flow measurements in a physical model of NPT‐assisted anesthesia. We assessed the accuracy of this method by comparing measured and calculated oropharyngeal pressure values and verified its feasibility in young children undergoing NPT‐assisted anesthesia for diagnostic endoscopy.

## 2 METHODS

### 2.1 Experimental study

The method we used for calculating oropharyngeal pressure from ΔP_NPT_ involved 3 subsequent methodological steps: (a) measuring the flow‐dependent ΔP across an NPT and interpolating the ΔP‐flow relationship with Rohrer's equation; (b) calculating the flow‐dependent ΔP across the NPT in simulated clinical scenarios using Rohrer's coefficients from the previous step, and (c) calculating oropharyngeal pressure from Paw and ΔP in the simulated scenarios.

#### 2.1.1 Step‐1: Pressure drop‐flow relationship for an NPT open to the atmosphere

**FIGURE 1:**
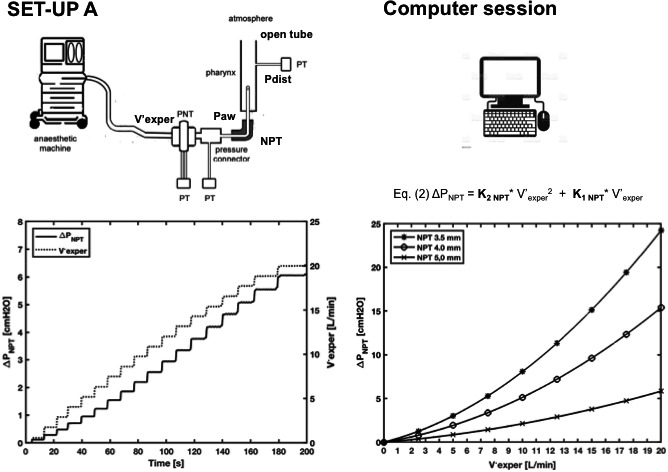
An anesthetic machine is used to deliver the experimental flow (V′_exper_) in various gas compositions (oxygen in this example) through a nasopharyngeal tube (NPT) inserted in an open tube. V′_exper_ is measured using a pneumotachograph (PNT) connected to a pressure transducer (PT); NPT proximal airway pressure (Paw) is measured through the lateral wall of a pressure connector connected to a PT and distal pressure (Pdist) is measured distal to the NPT tip through the lateral wall of the open tube. Set‐up A, left graph: ΔPNPT (Paw minus Pdist) and V′_exper_ are acquired over 200 s, using a 5.0 mm diameter NPT (in this example). V′_exper_ stepwise acquisition yields a quasi‐stationary flow (QSF). Computer session, right graph: ΔPNPT is plotted against V′_exper_ and interpolated with Rohrer's equation. The experiment is repeated for the three NPTs (3.5, 4.0, and 5.0 mm in diameter)

In step‐1, we measured the flow‐dependent ΔP across three NPTs open to atmosphere and then interpolated the ΔP‐flow relationships with Rohrer's equation to extrapolate the equation's coefficients needed for step‐2. This step involved an experimental setup (set‐up A) (Figure 1, left panel) and a computer session (Figure 1, right panel). Set‐up A was used to measure flow (experimental flow, V′_exper_) and ΔP across three NPTs (3.5, 4.0, and 5.0, mm inner diameter, the most commonly used in pediatrics) open to the atmosphere. V′_exper_ was pure oxygen or oxygen mixed with sevoflurane at different concentrations (oxygen, 2% sevoflurane in oxygen [2%sevo/O_2_], 4% sevoflurane in oxygen [4%sevo/O_2_], 6% sevoflurane in oxygen [6%sevo/O_2_], and 8% sevoflurane in oxygen [8%sevo/O_2_]). V′_exper_ was delivered up to 20 L/min (1 L/min step) in a way that resembled flow through the NPT during NPT‐anesthesia, that is, a continuous flow component (V′_NPT_) and a sinusoidal component (patient's breathing) combined (quasi‐stationary flow, QSF).^9^ ΔP_NPT_ was measured simultaneously with V′_exper_.

In the computer session, ΔP_NPT_ and V′_exper_ measured in set‐up A for each of the three NPTs were plotted one against the other, yielding three curves that were approximated by Rohrer's equation.^10^

(1) $$\Delta P=K_{2}\times V^{\prime 2}+K_{1}\times V^{\prime }$$

Substituting ΔP in Rohrer's equation with ΔP_NPT_ and V′ with V′_exper_ yielded (Figure 1, right panel)

(2) $${\Delta P}_{\text{NPT}}=K_{2\text{NPT}}\times V_{\text{exper}}^{\prime 2}+K_{1\text{NPT}}\times V_{\text{exper}}^{\prime }$$

For Figure 1, only oxygen flow was used. Figure S1 shows all the five gas combinations.

#### 2.1.2 Step‐2: Pressure drop‐flow relationship for the NPT in simulated clinical scenarios

**FIGURE 2:**
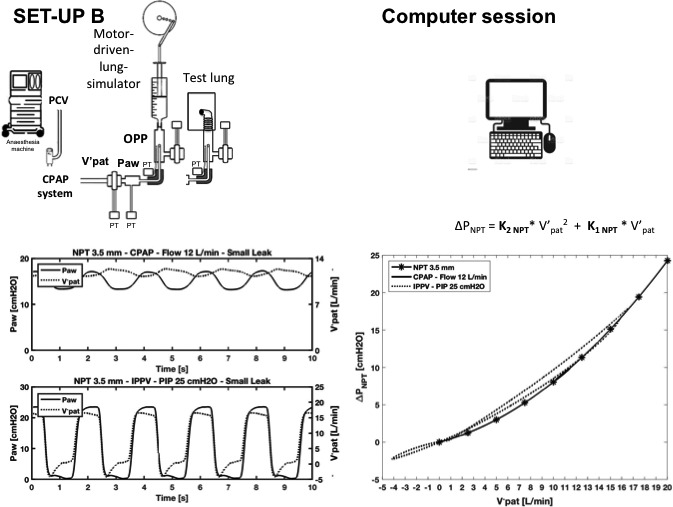
A motor‐driven lung simulator is used to simulate the spontaneous sinusoidal breathing during continuous positive airway pressure (CPAP) and a test lung is used during intermittent positive pressure ventilation (IPPV). IPPV is delivered by setting the ventilator in pressure‐controlled ventilation (PCV) with a peak inspiratory pressure (PIP) of 25 cmH_2_O, whereas for CPAP, a system connected to the anesthetic machine is used and set at 12 L/min (in this example). Flow is driven through a 3.5 mm diameter nasopharyngeal tube (NPT) inserted into an artificial pharynx with an opening simulating an open mouth. Set‐up B, left graph, shows proximal airway pressure (Paw) and flow through the NPT (V′_pat_) during CPAP or IPPV over time. Computer session, the two ΔPNPT during CPAP and IPPV are calculated by replacing V′_exper_ with the two V′_pat_ into Rohrer's equation. Right graph shows Rohrer's interpolated curve for the 3.5 mm NPT obtained in the computer session (Figure 1), and the two ΔPNPT and V′_pat_ relationships (dotted and thick lines). Both these curves lie on Rohrer's interpolated curve [Colour figure can be viewed at wileyonlinelibrary.com]

In step‐2, we calculated ΔP across each of the three NPTs in various clinical scenarios using Rohrer's equation, the coefficients obtained in step‐1, and flow delivered in the simulated scenarios. This step involved an experimental setup (set‐up B) and a computer session. The experimental setup shown in Figure 2 (left panel) was used to measure Paw and flow in two representative clinical scenarios: a patient receiving 3.5 mm NPT‐assisted anesthesia under CPAP at 12 L/min, or under IPPV at peak inspiratory pressure (PIP) of 25 cmH_2_O. In this step, unlike step‐1, flow combined V′_NPT_ and patient's breathing flow. We refer to this flow briefly as V′_pat_. Technical details on ΔP _NPT_ and flow measurements are provided in Appendix S1. The computer session (Figure 2, right panel) was used to calculate ΔP_NPT_. To do so, V′_pat_ substituted V′_exper_ in the Equation (2); thus, ΔP_NPT_ = K_2 NPT_ × V′_pat_^2^ + K_1 NPT_ × V′_pat_, whereas K_1_ and K_2_ were those obtained in step‐1 for each tube size and each gas composition.

For subsequent comparisons between calculated and measured oropharyngeal pressure, we simulated various clinical scenarios, which included differently aged children undergoing NPT‐assisted anesthesia using 3.5, 4.0, or 5.0 inner diameter tubes, under two degrees of mouth opening (large leak, inner diameter 4.0 mm, and small leak, 2.5 mm). Leaks were sized in preliminary experiments, using a 4 mm inner diameter NPT, aiming at a maximum oropharyngeal pressure under CPAP of around 3‐8 cmH_2_O for small leaks and 1‐3 cmH_2_O for large leaks depending on delivered flow rate. We simulated two respiratory conditions: spontaneous breathing under CPAP at 30 breaths per minute and 50 ml tidal volume and three delivered flow (5, 8, and 12 L/min), and apnea condition assisted by IPPV with two peak inspiratory pressure levels (15 and 25 cmH_2_O), zero PEEP, inspiratory time 1 s, 30 breaths/min, set flow 5 L/min. CPAP was delivered using a modified T‐tube with an adjustable resistance connected to the anesthetic machine (Primus) for delivering oxygen and oxygen mixed with sevoflurane through the NPT and thus modify pressure levels (the expiratory resistance was kept constant). We also simulated IPPV by setting the ventilator on the anesthetic machine in the pressure control ventilation mode (PCV). To detect the pressure and flow signals needed to calculate oropharyngeal pressure, we inserted the distal NPT in an artificial pharynx connected either to a motor‐driven lung‐model^11^ used as a pump to simulate sinusoidal breathing flow during CPAP or to a test lung (Quick lung junior, IngMar, Medical, 10 ml/cmH_2_O compliance, and 5 cmH_2_O /L/s resistance) during IPPV (Figure 2, set‐up B, left panel). The artificial pharynx had an opening that simulated two degrees of mouth opening (large leak, inner diameter 4.0 mm, and small leak, 2.5 mm). Leaks under IPPV were defined as volume through the NPT during inspiration minus lung simulator volume divided by volume through the NPT during inspiration and multiplied by 100. Large leaks were as high as 98%, whereas small leaks were around 60%.

All the experiments were repeated using oxygen and oxygen plus sevoflurane (oxygen, 2%sevo/O_2_, 4%sevo/O_2_).

#### 2.1.3 Step‐3: Calculating oropharyngeal pressure in the simulated clinical scenarios

**FIGURE 3:**
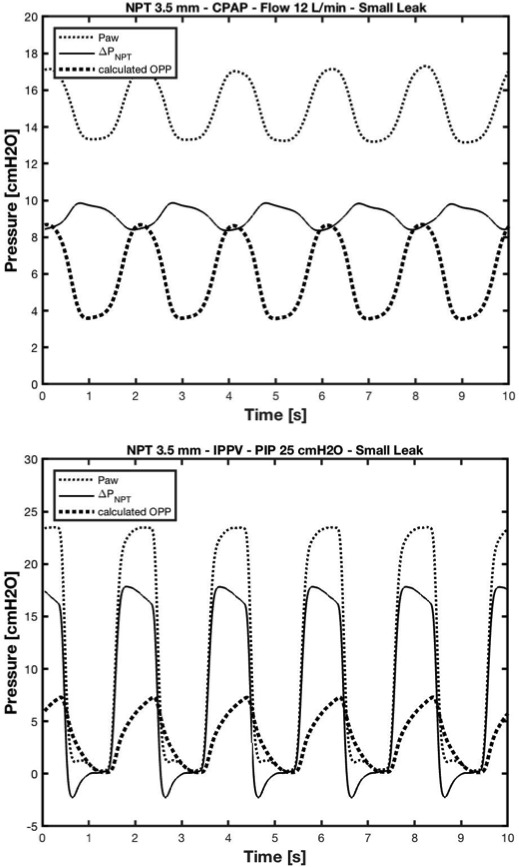
Upper graph shows proximal airway pressure (Paw), flow‐dependent pressure drop (ΔPNPT) and oropharyngeal pressure (OPP) over time during continuous positive pressure ventilation (CPAP) at 12 L/min using a 3.5 mm NPT. Lower graph shows Paw, ΔPNPT and OPP over time during intermittent positive pressure ventilation (IPPV), peak inspiratory pressure (PIP) of 25 cmH_2_O, using a 3.5 mm NPT. A point to point subtraction of ΔPNPT from Paw yielded OPP

In step‐3, we calculated oropharyngeal pressure in the simulated clinical scenarios as Paw minus ΔP _NPT_ computed in step‐2. In Figure 3 are shown Paw, ΔP_NPT_, and calculated oropharyngeal pressure tracings of the two representative clinical scenarios.

### 2.2 Accuracy of calculated oropharyngeal pressure

To determine the accuracy of calculated oropharyngeal pressure, we calculated and directly measured oropharyngeal pressure in the simulated clinical scenarios previously described.

Oropharyngeal pressure was directly measured in the pharynx with a thin catheter (Figure 2, left panel), with a closed tip and a lateral opening, connected to a pressure transducer (SensorTechnics 144SM070D‐PCB, SensorTechnics, Inc.). Maximum and minimum measured and calculated oropharyngeal pressure values were compared.

### 2.3 Clinical studies

The study received hospital institutional review board approval. Informed consent was obtained from parents or guardians. Oropharyngeal pressure, Paw, and flow data were obtained from patients younger than 4 years undergoing NPT‐assisted anesthesia for digestive or airway endoscopy. Five patients received inhalation anesthesia with 4% sevoflurane in pure oxygen (8% at induction). A 4 mm inner diameter NPT (Mallinckrodt™; Covidien) was inserted through the nostril and fixed with the tip 0.5‐1 cm above the vocal cords. A pneumotachograph and a pressure connecting tube were inserted proximally to the NPT. A second catheter (3 mm external diameter) was inserted into the pharynx through the other nostril. The final catheter position was checked by direct vision when endoscopy began. Variables were measured before endoscopy. The pressure‐flow relationship across the NPT for K_2_ and K_1_ determination was characterized using a tube of the same size from the same manufacturer.

### 2.4 Analysis

We used the goodness‐of‐fit test (MATLAB^®^ software, version 9.0.0.341360 [R2020b]) to measure how well the measured pressure and flow relationships fitted the interpolated curve built with Rohrer's equation. To verify the accuracy of oropharyngeal pressure calculated curves, we determined the root mean square deviation (RMSD) between measured and calculated values. For comparisons, we considered maximum and minimum oropharyngeal pressure values. Results were defined as satisfactory if RMSD was less than 0.5 cmH_2_O, and acceptable if it was less than 1 cmH_2_O. One‐way analysis of variance (ANOVA) was used to compare pressure values obtained at various gas compositions. Values are shown as mean (standard deviation, SD) or otherwise stated. A *p* value less than .05 was considered to indicate statistical significance.

## 3 RESULTS

### 3.1 Experimental study

#### 3.1.1 ΔP_NPT_/V′_exper_ relationships fitted by Rohrer's equation

Plotting ΔP_NPT_ for the three NPTs (3.5, 4 and 5 mm) against V′_exper_ under the five gas compositions yielded five distinct curves for each tube, which Rohrer's equation fitted with a high level of agreement (GOF ≈ 1, Table 1, Figure S1). The fitted curves yielded different slopes, and slopes steepened as the NPT diameters decreased and sevoflurane percentages increased. Accordingly, Rohrer's coefficients K_2_ increased with decreasing tube size and increasing sevoflurane concentrations (Table 1) (*p* < .0001 ANOVA).

**TABLE 1:** Rohrer's coefficients K_2_ and K_1_ of the interpolated pressure‐flow relationships of 3.5, 4.0, and 5.0 mm inner diameter nasopharyngeal tubes (NPTs) using oxygen (O_2_) and the four sevoflurane (sevo) combinations

NPT	Gas composition	K_1_ [cmH_2_O × s/L]	K_2_ [cmH_2_O × s^2^/L^2^]	GOF
Diameter		Mean (CI)	Mean (CI)	Mean (CI)
3.5 mm	O_2_	17.6 (17.5‐17.7)	175.1 (175.0‐175.2)	0.99 (0.99‐0.99)
	2%sevo/O_2_	18.8 (18.7‐18.9)	200.3 (200.2‐200.4)	0.99 (0.99‐0.99)
	4%sevo/O_2_	22.1 (22.0‐22.2)	215.5 (215.4‐215.6)	0.99 (0.99‐0.99)
	6%sevo/O_2_	24.2 (24.1‐22.3)	218.7 (218.6‐218.8)	0.99 (0.99‐0.99)
	8%sevo/O_2_	29.5 (29.4‐29.6)	219.6 (219.5‐219.7)	0.99 (0.99‐0.99)
4.0 mm	O_2_	16.9 (16.8‐17.0)	86.3 (86.2‐86.4)	0.99 (0.99‐0.99)
	2%sevo/O_2_	13.3 (13.2‐13.4)	115.8 (115.7‐115.9)	0.99 (0.99‐0.99)
	4%sevo/O_2_	14.6 (14.5‐14.7)	126.8 (126.7‐126.9)	0.99 (0.99‐0.99)
	6%sevo/O_2_	14.4 (14.3‐14.5)	131.4 (131.3‐119.5)	0.99 (0.99‐0.99)
	8%sevo/O_2_	18.2 (18.1‐18.3)	131.9 (131.8‐132.0)	0.99 (0.99‐0.99)
5.0 mm	O_2_	7.9 (7.8‐8.0)	29.0 (28.9‐29.1)	0.99 (0.99‐0.99)
	2%sevo/O_2_	9.0 (8.9‐9.1)	30.3 (30.2‐30.4)	0.99 (0.99‐0.99)
	4%sevo/O_2_	9.7 (9.6‐9.8)	31.6 (31.5‐31.7)	0.99 (0.99‐0.99)
	6%sevo/O_2_	9.8 (9.7‐9.9)	33.0 (32.9‐33.1)	0.99 (0.99‐0.99)
	8%sevo/O_2_	10.3 (10.2‐10.4)	34.2 (121.1‐121.3)	0.99 (0.99‐0.99)

Note

Level of agreement between experimental and interpolated curves was calculated by the goodness‐of‐fit (GOF) test.

Abbreviation: CI, confidential intervals.

#### 3.1.2 ΔP_NPT_ changes related to NPT size, flow, sevoflurane, Paw, and leaks in the simulated clinical scenarios

During NPT‐simulated anesthesia in the CPAP modality, ΔP_NPT_ mean values markedly increased with flow and the smaller NPT sizes. Although the tested sevoflurane concentrations (2% and 4%) were lower than all of those potentially used in clinical practice, they still yielded an appreciable increase in ΔP_NPT_ mean values with increasing concentrations, especially at higher flow rates (Table S1). Similarly, Paw mean values increased with increasing flow and sevoflurane (Table S1). Because the flow generator we used for CPAP was not “ideal,” leaks slightly increased flow through the NPT and thus increased the pressure drop (Table S1).

During NPT‐simulated anesthesia in the IPPV modality, ΔP_NPT_ mean values increased with increasing Paw (15 cmH_2_O vs 25 cmH_2_O) and the smaller NPT sizes; large leaks also markedly increased ΔP_NPT_ (Table S2).

#### 3.1.3 Oropharyngeal pressure changes related to NTP size, flow, sevoflurane, Paw, and leaks in the simulated clinical scenarios

In our flow‐regulated CPAP system, mean oropharyngeal pressure values increased with flow and sevoflurane concentrations, but were unaffected by NPT size (Table S1). During IPPV, oropharyngeal pressure increased with increasing NPT size and Paw, whereas it decreased with increasing leak size (Table S2).

### 3.2 Oropharyngeal pressure calculation accuracy (RMSD)

Oropharyngeal pressure calculated in the clinical scenarios using the coefficients K_2_ and K_1_ generated using V′_exper_ approximated the measured oropharyngeal pressure well, as demonstrated by satisfactory RMSDs (<0.5 cmH_2_O) between calculated and measured maximum and minimum values during CPAP or IPPV (Tables S1 and S2). Oropharyngeal pressure calculation accuracy slightly decreased with increasing flow and sevoflurane concentrations although RMSDs remained below 0.5 cmH_2_O (Tables S1 and S2).

### 3.3 Clinical studies

**FIGURE 4:**
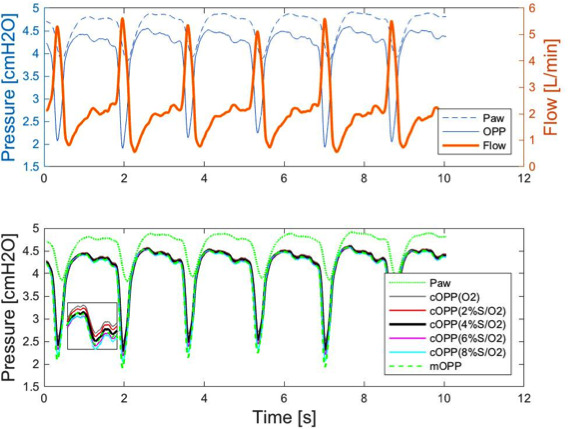
Upper panel: airway pressure (Paw), oropharyngeal pressure (OPP), and flow tracings in a young child undergoing nasopharyngeal tube‐assisted anesthesia with 4% sevoflurane in oxygen (S/O_2_); lower panel: tracings of Paw, measured OPP (mOPP) and calculated OPP (cOPP) using oxygen (O_2_) and the four sevoflurane combinations [Colour figure can be viewed at wileyonlinelibrary.com]

In the five infants undergoing NPT‐assisted anesthesia for endoscopy with 4%sevo/O_2_ (8% at induction), the calculated and measured oropharyngeal pressure curves were close. Considering the five patients overall, RMSDs were 0.19 (0.10) cmH_2_O for maximum values and 0.49 (0.18) cmH_2_O for minimum values. A typical example of measured and calculated oropharyngeal pressure curves in a 1‐year‐old infant breathing 8%sevo/O_2_ during the induction phase yielded RMSDs of 0.021 cmH_2_O for maximum and 0.34 cmH_2_O for minimum and 0.05 cmH_2_O for maximum and 0.30 cmH_2_O for minimum during the maintenance phase (4%sevo/O_2_) (Figure 4).

## 4 DISCUSSION

These results show that the stepwise approach we used for characterizing ΔP_NPT_ makes it easy to calculate oropharyngeal pressure in a clinical scenario of NPT‐anesthesia using Rohrer's equation and its coefficients. This method proves accurate regardless of NPT size, flow, Paw, leak size, and gas composition. We also confirmed the method's feasibility in children undergoing NPT‐assisted anesthesia for endoscopic procedures.

### 4.1 Mathematical model

To estimate oropharyngeal pressure under NPT‐assisted anesthesia, we used the previously tested mathematical model elaborated by Guttman et al^8^ for calculating intra‐tracheal pressure in mechanically ventilated adults and children. The clinical setting in which we applied Guttman's mathematical construct nevertheless differs from their previous studies in several aspects. First, we applied this method in NPT‐assisted anesthesia, a clinical scenario that can involve exceedingly large leaks. Second, we considered spontaneous breathing assisted by CPAP, a clinical scenario that differs from positive pressure mechanical ventilation in terms of pressure and flow profiles. Finally, our scenario included anesthetized patients and, thus, a potentially different inhaled gas composition.

Despite these premises, we decided to test this mathematical model unchanged. We took this decision based on previous studies on intra‐tracheal pressure calculated using uncuffed tubes with moderate flow leakage around the tube, in which the authors reported RMSDs less than 0.5 cmH_2_O between coupled, calculated, and measured intra‐tracheal pressure values.^12^ The results of our study proved our choice correct, given that we found an excellent agreement between measured and calculated coupled oropharyngeal pressure values. Indeed, variations in NPT size, gas composition, flow rate, pressure applied to the airways, and leaks from the upper airways left oropharyngeal pressure nearly unchanged in accuracy, as the low RMSD values confirmed (Tables S1 and S2). Our findings agree with previous observations by Hentschel et al, who investigated how ventilator settings influenced tracheal pressure swings in an infant mechanical ventilation model. Although respiratory variables affected ΔP_NPT_ entity by altering flow delivery (as it should from a physical point of view), oropharyngeal pressure calculation remained unchanged in accuracy.^13^ In our study, the only factors that increased the differences between calculated and measured oropharyngeal pressure values were the highest flow and sevoflurane concentrations, although oropharyngeal pressure calculation remained satisfactory even in the in vivo studies. Unlike the respiratory variables that impact ΔP_NPT_ by changing gas flow velocity, the sevoflurane‐induced changes in ΔP_NPT_ depend on the gas density. The viscosity and density of a gas flowing through a tube strongly affect the pressure‐flow relationship across the NPT and thus K_2_ in Rohrer's equation.^14^ In our study, K_2_ increased by up to 50% at higher sevoflurane concentrations. Increased NPT resistance related to increased K_2_ explains why Paw increased with sevoflurane, although a higher CPAP‐expiratory valve resistance caused by sevoflurane may have contributed. Unexpectedly, oropharyngeal pressure also increased. If we assume that an ideal channel between the pharynx and the mouth allows flow heading toward the mouth to escape, we understand why a denser gas composition by exerting higher resistance increases the proximal pressure, that is, oropharyngeal pressure.

Our clinical scenario of NPT‐anesthesia proved particularly suitable for calculating oropharyngeal pressure. Owing to the leaks, a continuous flow crosses the NPT; thus, the patient's respiratory flow oscillates over a continuous positive flow. For this reason and because some expiratory flow leaks from the mouth, the expiratory flow tracing rarely crosses the zero‐flow line and becomes negative (Figure 4). Hence, the negative flow coefficients of the pressure‐flow relationship across the NPT (−K_2_)^15^ are hardly needed. Also, the continuous flow helps smooth flow inversion between inspiration and expiration, thus preventing inertia and hysteresis, complications that our experiments rarely induced but Guttman often had to cope with.^12^

Although we used the same mathematical model as Guttman et al did in their studies, our coefficients differ from those previously reported.^16^ These discrepancies probably reflect the different tube curvature during the pressure‐flow characterization and, most importantly, the quasi‐stationary flow that we used instead of the sinusoidal flow used in previous studies.

### 4.2 In vivo studies

Oropharyngeal pressure calculation provided higher RMSDs in vivo than in in vitro experiments. In all patients studied, the maximum and minimum measured oropharyngeal pressure values were lower than the calculated ones, suggesting that a higher tube resistance in vivo increased the pressure drop, a condition that in vitro data might find it difficult to predict. These findings are in line with those previously reported by Wright et al,^17^ who found that in vivo endotracheal tube airflow resistance is often significantly higher than indicated in vitro. As contributory mechanisms for this discrepancy in vivo, they suggested probable tube obstruction or tracheal wall impingement. A further explanation might be the different flow pattern that we observed between spontaneous and simulated breathing. The impact of flow and pressure waveforms on the pressure drop across the tube has been previously investigated by Hentschel et al, who showed that in intubated small infants, ventilator settings leading to high gas flow velocity predisposed to a high‐pressure drop across the tube. In our study, the sharper flow waves observed in vivo versus in vitro and the profound inspiratory pressure waves suggest higher flow velocities along the NPT and, consequently, increased flow resistance.^13^

### 4.3 Clinical considerations

As pediatric procedural sedations continue to increase in number, having a “tubeless” technique that can provide oxygen, anesthetics and possibly emergency ventilation would help improve patients' safety and operators' satisfaction. Once popular among anesthesiologists, the NPT has become less interesting over the years, possibly because no tools have been available to assess its physiologic effects. In this study, we have described a simple effective method to calculate oropharyngeal pressure during NPT‐anesthesia using a flow and a pressure sensor connected to the proximal end of the NPT and applying specific mathematical algorithms. Although it is unlikely that commercial ventilators will soon include a program to calculate oropharyngeal pressure using our or other's methods, our oropharyngeal pressure monitoring system remains a potentially useful research tool. Indeed, research is needed to verify whether NPT ventilation delivers a therapeutic breathing pressure in clinical practice. Besides, if pharyngeal pressure is known, mathematical algorithms might be implemented from pressure data to calculate flow leak volume and thus tidal volume during NPT ventilation. This method would be of great help when the airway opening and the operative field are shared and spirometry is unpractical. In a similar investigation, in a setting involving mechanical ventilation with tube leaks, Nikischin and Lange sought to find the leak volume and did so using the proportionality with airway pressure and its duration of application.^18^

**FIGURE 5:**
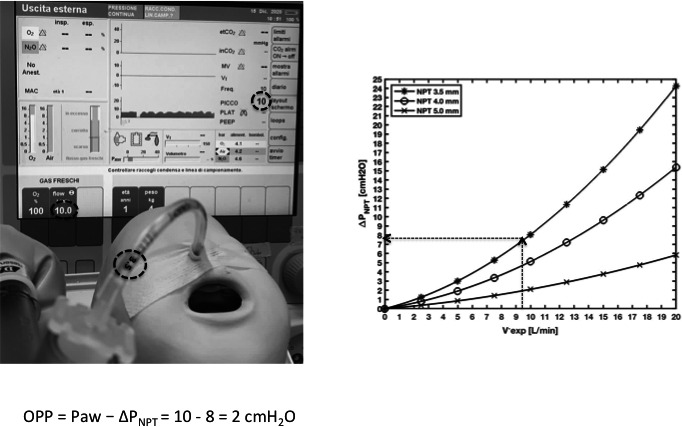
A simulated infant undergoing nasopharyngeal tube (NPT)‐continuous positive airway pressure (CPAP) with a 3.5 mm tube, through an anesthesia bag with the expiratory valve kept almost closed and connected to an anesthetic machine. Flow is set at 10 L/min. Paw is read on the screen. ΔPNPT is assessed by drawing a vertical line from 10 L/min on the *X*‐axis to intercept the curve and then a horizontal line on the *Y*‐axis shows ΔPNPT. Oropharyngeal pressure (OPP) is calculated as Paw minus ΔPNPT

Other than providing an exploratory glance at the research value of our oropharyngeal pressure calculation method, we try to offer physicians some clinical clues that could help them to determine oropharyngeal pressure in their daily practice using the measurements they have on hand. A common way to deliver oxygen through an NPT is to use a flow‐inflating bag because it allows patients to breathe either spontaneously or passively if the anesthesiologist squeezes the bag. In addition, the bag makes it easier to feel lung compliance, respiratory effort, rate, and tidal volume than do other systems including the ventilator. By adjusting the expiratory valve, a flow‐inflating bag also provides variable positive end‐expiratory pressure. The flow‐inflating bag connected to the anesthetic machine allows anesthesiologists to deliver oxygen and anesthetics and read Paw and flow on the monitor. Because Paw overestimates oropharyngeal pressure for ΔP_NPT_ (as long as flow remains positive), to assess ΔP_NPT_, we suggest choosing the pressure‐flow curve (Figure S1) corresponding to the NPT diameter and gas composition used in the clinical setting. Then, by drawing a vertical line from the set flow value on the *X*‐axis to intercept the curve, we obtain the corresponding ΔP_NPT_ on the *Y*‐axis. ΔP_NPT_ will then be subtracted from Paw. For example, if we are using a 3.5 mm NPT and 8 L/min oxygen flow rate, if the expiratory valve is almost closed, by plotting 8 L/min on the pressure drop/flow curve for the 3.5 mm NPT, we can approximate ΔP_NPT_. Once ΔP_NPT_ is obtained, we can subtract it from Paw and get oropharyngeal pressure (Figure 5). Otherwise, the mean flow seen on the monitor can be plotted to estimate the mean pressure drop.

Following this practice, our residents are no longer worried about using high Paw values during NPT ventilation in emergency situations especially when the tube diameter is small, because they know that ΔP_NPT_ is probably high and the resulting oropharyngeal pressure will therefore be far below the harm threshold. Similarly, when anesthesia is induced in difficult‐to‐intubate patients after carefully titrating sevoflurane in oxygen to maintain spontaneous respiration, having specific sevoflurane pressure‐flow curves could help determine ΔP_NPT_ more precisely using its homologous curve (Figure S1). When using sevoflurane, the low flow we use to reduce the scavenging problem often elicits barely appreciable pharyngeal pressure probably unable to split the airway. In this situation, airway patency is better maintained using the jaw thrust maneuver throughout the procedure.

This study has limitations. We investigated few subjects with limited breathing patterns. A larger and more clinically diverse sample of patients should be studied to further validate whether our method for oropharyngeal pressure calculation covers the significant variability in breathing patterns that exists in pediatric ventilation. In patients who suffer from labored breathing, calculated and measured oropharyngeal pressure are more likely to differ because we characterized the pressure‐flow relationship with the quasi‐stationary flow, a method that might be unsuitable for marked respiratory flow accelerations and decelerations in a patient awake or mildly sedated. Another limitation is that our simplified rigid pharyngeal model differed from a model using normal tissue. This drawback prevented us from investigating how pharyngeal compliance influenced oropharyngeal pressure calculation. Our method's accuracy should remain uninfluenced by compliance at the end of the NPT because ΔP_NPT_ reflects only the NPT characteristics and flow delivered through the tube. Nevertheless, compliance might possibly change oropharyngeal pressure values depending on pressure, but if it did, these changes would depend on variations in gas flow velocity. Finally, our method's reliability depends on NPT patency. If the tube is obstructed, Paw becomes far higher than expected for the delivered flow, so that this discrepancy may be seen as a warning sign for NPT obstruction.

In conclusion, during NPT‐assisted anesthesia, Paw readings on the anesthetic monitoring machine overestimate oropharyngeal pressure especially for smaller‐diameter NPTs and higher flow, and to a lesser extent for large leaks. Given its clinical importance in guiding NPT ventilation in young children, we propose an accurate method for calculating oropharyngeal pressure using Rohrer's equation, or approximating clinically monitored flow and pressure values using ΔP_NPT_‐flow relationship charts.

## CONFLICT OF INTEREST

The authors declare that they have no conflict of interest.

## Supporting information

Figure S1

Table S1

Table S2

Appendix S1

## Data Availability

The data that support the findings of this study are available from the corresponding author upon reasonable request.
