# Supplementary figures and images for: Nasopharyngeal tubes in pediatric anesthesia: Is the flow‐dependent pressure drop across the tube suitable for calculating oropharyngeal pressure?
Source: Paediatr Anaesth. 2021 May 6;31(7):809–19. doi: 10.1111/pan.14194 (PMC8252547; doi:10.1111/pan.14194)

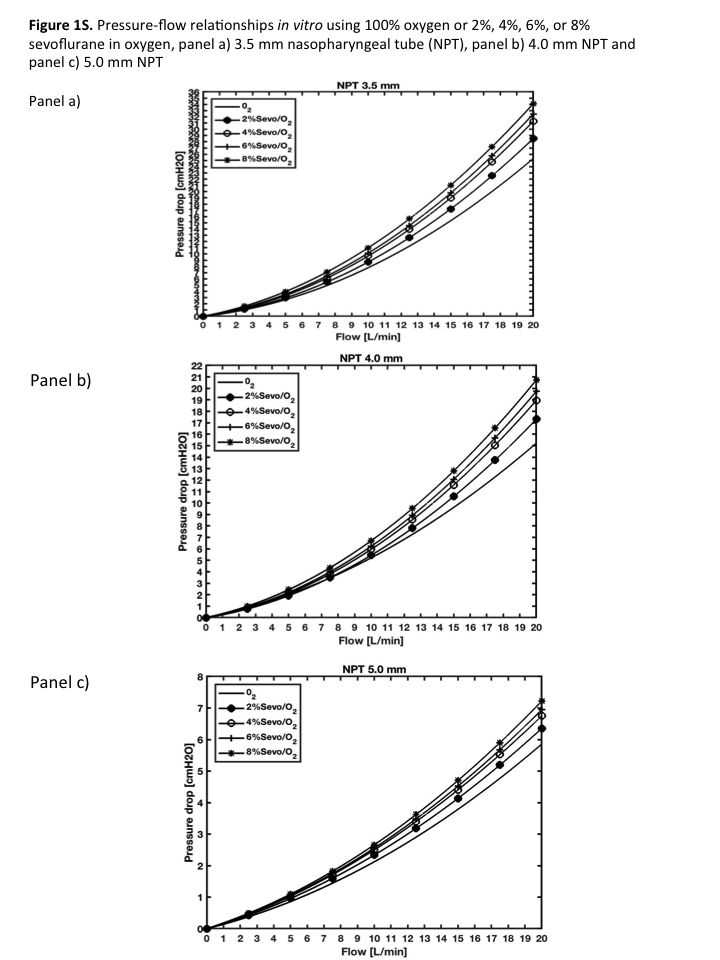

Supplement: Supplementary file 1 — Figure S1 [file PAN-31-809-s004.tiff]
