# Supplementary material for: Nasopharyngeal tubes in pediatric anesthesia: Is the flow‐dependent pressure drop across the tube suitable for calculating oropharyngeal pressure?
Source: Paediatr Anaesth. 2021 May 6;31(7):809–19. doi: 10.1111/pan.14194 (PMC8252547; doi:10.1111/pan.14194)
Supplement: Supplementary file 2 — Table S1 [file PAN-31-809-s003.docx]

**Table 1S** Proximal airway pressure (Paw), calculated oropharyngeal pressure (OPP) and pressure drop (∆P) mean values. Root mean square deviation (RMSD) mean values between calculated and measured OPP under continuous positive airway pressure (CPAP) at different set flow rates, gas composition and leak size. For Paw and OPP maximum (max) and minimum (min) mean values are given. For ∆P max and min values are opposites.

|  |  | **Paw/OPP/∆P** | | | **RMSD** | | | | |  | **Paw/OPP/∆P** | | | **RMSD** | | | | |
| --- | --- | --- | --- | --- | --- | --- | --- | --- | --- | --- | --- | --- | --- | --- | --- | --- | --- | --- |
| **NPT** | **Value** | **O_2_** | **2%Sevo/O_2_** | **4%Sevo/O_2_** | **O_2_** | **2%Sevo/O_2_** | | **4%Sevo/O_2_** | |  | **O_2_** | **2%Sevo/O_2_** | **4%Sevo/O_2_** | **O_2_** | **2%Sevo/O_2_** | | **4%Sevo/O_2_** | |
| **size** |  | **[cmH_2_O]** | | |  | **[cmH_2_O]** | |  | |  |  | **[cmH_2_O]** | | **[cmH_2_O]** | | | | |
|  |  | **Flow 5 L/min - small leaks** | | | | | | | |  | **Flow 5 L/min - large leaks** | | | | | | | |
| **3.5 mm** | **Max** | **6.6/3.3/3.3** | **6.9/3.4/3.5** | **7.1/3.6/3.5** | **0.02** | | **0.08** | | **0.07** |  | **3.8/0.8/3.0** | **4.1/0.9/3.2** | **4.5/1.0/3.5** | **0.03** | | **0.06** | | **0.08** |
| **3.5 mm** | **Min** | **3.5/0.2/3.3** | **3.8/0.3/3.5** | **4.2/0.6/3.6** | **0.03** | | **0.07** | | **0.02** |  | **3.2/0.1/3.1** | **3.6/0.1/3.5** | **3.9/0.1/3.8** | **0.02** | | **0.05** | | **0.06** |
|  |  | **Flow 8 L/min - small leaks** | | | | | | | |  | **Flow 8 L/min - large leaks** | | | | | | | |
| **3.5 mm** | **Max** | **10.3/6.3/4.0** | **11.2/6.8/4.4** | **12.3/7.4/4.9** | **0.06** | | **0.02** | | **0.05** |  | **7.1/1.4/5.7** | **7.8/1.5/6.3** | **8.4/1.7/6.7** | **0.07** | | **0.09** | | **0.10** |
| **3.5 mm** | **Min** | **7.3/1.5/5.8** | **8.1/1.8/6.3** | **8.8/1.9/6.9** | **0.03** | | **0.06** | | **0.04** |  | **6.3/0.3/6.0** | **6.9/0.4/6.5** | **7.5/0.5/7.0** | **0.13** | | **0.12** | | **0.15** |
|  |  | **Flow 12 L/min - small leaks** | | | | | | | |  | **Flow 12 L/min - large leaks** | | | | | | | |
| **3.5 mm** | **Max** | **17.5/8.8/8.7** | **19.1/9.8/9.3** | **20.5/10.5/10.0** | **0.12** | | **0.15** | | **0.18** |  | **12.2/2.2/10.0** | **13.2/2.3/10.9** | **14.3/2.6/11.7** | **0.13** | | **0.11** | | **0.13** |
| **3.5 mm** | **Min** | **13.1/3.5/9.6** | **14.6/4.0/10.6** | **15.8/4.4/11.4** | **0.14** | | **0.13** | | **0.17** |  | **11.0/0.9/10.1** | **12.1/1.0/11.1** | **13.0/1.1/11.9** | **0.02** | | **0.01** | | **0.03** |
|  |  | **Flow 5 L/min - small leaks** | | | | | | | |  | **Flow 5 L/min - large leaks** | | | | | | | |
| **4.0 mm** | **Max** | **5.6/3.3/2.3** | **5.8/3.6/2.2** | **6.4/4.0/2.4** | **0.03** | | **0.06** | | **0.05** |  | **2.9/1.0/1.9** | **3.2/1.1/2.1** | **3.4/1.2/2.2** | **0.04** | | **0.03** | | **0.08** |
| **4.0 mm** | **Min** | **2.5/0.5/2.0** | **2.8/0.6/2.2** | **3.1/0.7/2.4** | **0.07** | | **0.00** | | **0.09** |  | **2.1/0.1/2.0** | **2.3/0.1/2.2** | **2.6/0.1/2.5** | **0.01** | | **0.02** | | **0.00** |
|  |  | **Flow 8 L/min - small leaks** | | | | | | | |  | **Flow 8 L/min - large leaks** | | | | | | | |
| **4.0 mm** | **Max** | **9.2/6.2/3.0** | **10.2/6.8/3.4** | **11.2/7.5/3.7** | **0.10** | | **0.25** | | **0.25** |  | **5.3/1.5/3.8** | **5.8/1.6/4.2** | **6.4/1.8/4.6** | **0.03** | | **0.04** | | **0.05** |
| **4.0 mm** | **Min** | **5.4/1.7/3.7** | **6.1/1.9/4.2** | **6.8/2.2/4.6** | **0.14** | | **0.11** | | **0.13** |  | **4.4/0.4/4.0** | **4.9/0.5/4.4** | **5.4/0.5/4.9** | **0.12** | | **0.12** | | **0.15** |
|  |  | **Flow 12 L/min - small leaks** | | | | | | | |  | **Flow 12 L/min - large leaks** | | | | | | | |
| **4.0 mm** | **Max** | **13.5/8.2/5.3** | **15.0/9.3/5.7** | **16.4/10.2/6.2** | **0.23** | | **0.27** | | **0.32** |  | **7.9/2.1/5.8** | **8.7/2.3/6.4** | **9.5/2.5/7.0** | **0.23** | | **0.29** | | **0.33** |
| **4.0 mm** | **Min** | **9.5/3.5/6.0** | **10.6/3.9/6.7** | **11.7/4.3/7.4** | **0.14** | | **0.17** | | **0.13** |  | **7.1/0.8/6.3** | **7.9/1.2/6.7** | **8.6/1.4/7.2** | **0.09** | | **0.10** | | **0.11** |
|  |  | **Flow 5 L/min - small leaks** | | | | | | | |  | **Flow 5 L/min - large leaks** | | | | | | | |
| **5.0 mm** | **Max** | **3.1/2.9/0.2** | **3.3/3.1/0.2** | **3.5/3.3/0.2** | **0.05** | | **0.08** | | **0.09** |  | **1.2/0.8/0.4** | **1.4/0.9/0.5** | **1.5/1.0/0.5** | **0.06** | | **0.09** | | **0.08** |
| **5.0 mm** | **Min** | **0.9/0.2/0.7** | **1.0/0.3/0.7** | **1.1/0.4/0.7** | **0.04** | | **0.08** | | **0.09** |  | **0.8/0.1/0.7** | **0.8/0.1/0.7** | **0.9/0.1/0.8** | **0.06** | | **0.09** | | **0.08** |
|  |  | **Flow 8 L/min - small leaks** | | | | | | | |  | **Flow 8 L/min - large leaks** | | | | | | | |
| **5.0 mm** | **Max** | **8.1/6.6/1.5** | **8.8/7.2/1.6** | **9.6/7.9/1.7** | **0.01** | | **0.02** | | **0.01** |  | **3.5/1.6/1.9** | **3.8/1.7/2.1** | **4.1/1.9/2.2** | **0.04** | | **0.02** | | **0.02** |
| **5.0 mm** | **Min** | **4.1/2.2/1.9** | **4.3/2.5/1.8** | **5.0/2.8/2.2** | **0.04** | | **0.03** | | **0.04** |  | **2.5/0.5/2.0** | **2.8/0.6/2.2** | **3.0/0.6/2.4** | **0.05** | | **0.03** | | **0.03** |
|  |  | **Flow 12 L/min - small leaks** | | | | | | | |  | **Flow 12 L/min - large leaks** | | | | | | | |
| **5.0 mm** | **Max** | **10.0/8.4/1.6** | **11.0/9.3/1.7** | **12.2/10.5/1.7** | **0.05** | | **0.04** | | **0.04** |  | **5.6/2.3/3.3** | **6.1/2.7/3.4** | **6.6/2.9/3.7** | **0.09** | | **0.09** | | **0.08** |
| **5.0 mm** | **Min** | **7.7/3.7/4.0** | **8.7/4.1/4.6** | **9.5/4.5/5.0** | **0.08** | | **0.08** | | **0.07** |  | **4.4/1.1/3.3** | **4.9/1.3/3.6** | **5.2/1.4/3.8** | **0.10** | | **0.10** | | **0.06** |
