# Supplementary material for: Nasopharyngeal tubes in pediatric anesthesia: Is the flow‐dependent pressure drop across the tube suitable for calculating oropharyngeal pressure?
Source: Paediatr Anaesth. 2021 May 6;31(7):809–19. doi: 10.1111/pan.14194 (PMC8252547; doi:10.1111/pan.14194)
Supplement: Supplementary file 3 — Table S2 [file PAN-31-809-s002.docx]

**Table 2S** Maximum and minimum proximal airway pressure (Paw), calculated oropharyngeal pressure (OPP) and pressure drop (∆P) mean values. Root mean square deviation (RMSD) mean values between calculated and measured OPP under intermittent positive pressure ventilation (IPPV) at different pressure levels, gas composition and leak size.

|  |  | **Paw/OPP/∆P** | | | | | | **RMSD** | | | | |  | **Paw/OPP/∆P** | | | | | **RMSD** | | | | |  |
| --- | --- | --- | --- | --- | --- | --- | --- | --- | --- | --- | --- | --- | --- | --- | --- | --- | --- | --- | --- | --- | --- | --- | --- | --- |
| **NPT** | **Value** | **O_2_** | **2%Sevo/O_2_** | | **4%Sevo/O_2_** | | | **O_2_** | **2%Sevo/O_2_** | | **4%Sevo/O_2_** | |  | **O_2_** | **2%Sevo/O_2_** | | **4%Sevo/O_2_** | | **O_2_** | **2%Sevo/O_2_** | | **4%Sevo/O_2_** | |  |
| **size** |  | **[cmH2O]** | | | | | | **[cmH2O]** | | | | |  | **[cmH2O]** | | | | | **[cmH2O]** | | | | |  |
|  |  | **Peak inspiratory pressure 15 cmH_2_O - small leaks** | | | | | | | | | | |  | **Peak inspiratory pressure 15 cmH_2_O - large leaks** | | | | | | | | | |  |
| **3.5 mm** | **Max** | **14.2/4.8/9.4** | | **14.2/4.8/9.4** | | | **14.2/4.8/9.4** | **0.02** | | **0.04** | | **0.05** |  | **14.1/1.8/12.3** | | **14.1/1.8/12.3** | | **14.0/1.8/12.2** | **0.01** | | **0.04** | | **0.06** |  |
| **3.5 mm** | **Min** | **0.3/0.1/0.2** | | **0.3/0.1/0.2** | | | **0.3/0.1/0.2** | **0.09** | | **0.11** | | **0.14** |  | **0.1/0.0/0.1** | | **0.1/0.0/0.1** | | **0.1/0.0/0.1** | **0.04** | | **0.04** | | **0.04** |  |
|  |  | **Peak inspiratory pressure 25 cmH_2_O - small leaks** | | | | | | | | | | |  | **Peak inspiratory pressure 25 cmH_2_O - large leaks** | | | | | | | | | |  |
| **3.5 mm** | **Max** | **23.5/7.3/16.2** | | **23.5/7.2/16.3** | | | **23.5/7.1/16.4** | **0.04** | | **0.04** | | **0.12** |  | **23.3/3.0/20.0** | | **23.3/3.1/20.2** | | **23.4/3.1/20.3** | **0.03** | | **0.03** | | **0.16** |  |
| **3.5 mm** | **Min** | **0.2/0.1/0.1** | | **0.3/0.1/0.2** | | | **0.3/0.1/0.2** | **0.01** | | **0.01** | | **0.01** |  | **0.1/0.0/0.1** | | **0.1/0.0/0.1** | | **0.1/0.0/0.1** | **0.10** | | **0.11** | | **0.12** |  |
|  |  |  | | | |  | | | | | | |  |  | | | | | | | | | |  |
|  |  | **Peak inspiratory pressure 15 cmH_2_O - small leaks** | | | | | | | | | | |  | **Peak inspiratory pressure 15 cmH_2_O - large leaks** | | | | | | | | | |  |
| **4.0 mm** | **Max** | **14.1/5.8/8.3** | | **14.1/5.5/8.6** | | | **14.1/5.6/8.5** | **0.02** | | **0.05** | | **0.15** |  | **14.2/2.4/11.8** | | **14.5/2.5/12.0** | | **14.6/2.5/12.1** | **0.07** | | **0.09** | | **0.15** |  |
| **4.0 mm** | **Min** | **0.3/0.1/0.2** | | **0.2/0.1/0.1** | | | **0.3/0.1/0.2** | **0.10** | | **0.13** | | **0.15** |  | **0.0/0.0/0.0** | | **0.1/0.0/0.1** | | **0.1/0.0/0.1** | **0.03** | | **0.03** | | **0.04** |  |
|  |  | **Peak inspiratory pressure 25 cmH_2_O - small leaks** | | | | | | | | | | |  | **Peak inspiratory pressure 25 cmH_2_O - large leaks** | | | | | | | | | |  |
| **4.0 mm** | **Max** | **23.2/8.2/15.0** | | **23.0/8.5/14.5** | | | **23.6/8.8/14.8** | **0.12** | | **0.21** | | **0.26** |  | **23.5/3.6/19.9** | | **23.5/3.8/19.7** | | **23.6/3.7/19.9** | **0.01** | | **0.10** | | **0.14** |  |
| **4.0 mm** | **Min** | **0.1/0.0/0.1** | | **0.0/0.0/0.0** | | | **0.0/0.0/0.0** | **0.03** | | **0.09** | | **0.04** |  | **0.0/0.0/0.0** | | **0.0/0.0/0.0** | | **0.0/0.0/0.0** | **0.03** | | **0.05** | | **0.04** |  |
|  |  |  | | | | | | | | | | |  |  | | | | | | | | | |  |
|  |  | **Peak inspiratory pressure 15 cmH_2_O - small leaks** | | | | | | | | | | |  | **Peak inspiratory pressure 15 cmH_2_O - large leaks** | | | | | | | | | |  |
| **5.0 mm** | **Max** | **14.3/9.2/5.1** | | **14.2/9.1/5.1** | | | **14.1/9.0/5.1** | **0.01** | | **0.20** | | **0.15** |  | **14.6/4.7/9.9** | | **14.7/4.7/10.0** | | **14.7/4.7/10.0** | **0.03** | | **0.15** | | **0.13** |  |
| **5.0 mm** | **Min** | **0.3/0.2/0.1** | | **0.3/0.2/0.1** | | | **0.3/0.2/0.1** | **0.02** | | **0.02** | | **0.01** |  | **0.0/0.0/0.0** | | **0.1/0.0/0.1** | | **0.1/0.0/0.1** | **0.03** | | **0.03** | | **0.04** |  |
|  |  | **Peak inspiratory pressure 25 cmH_2_O - small leaks** | | | | | | | | | | |  | **Peak inspiratory pressure 25 cmH_2_O - large leaks** | | | | | | | | | |  |
| **5.0 mm** | **Max** | **23.6/13.8/9.8** | | **23.5/13.6/9.9** | | | **23.5/13.6/9.9** | **0.03** | | **0.15** | | **0.07** |  | **23.0/7.7/15.3** | | **23.1/7.9/15.2** | | **23.2/7.7/15.5** | **0.17** | | **0.36** | | **0.36** |  |
| **5.0 mm** | **Min** | **0.9/0.9/0.0** | | **0.5/0.4/0.1** | | | **0.7/0.6/0.1** | **0.15** | | **0.09** | | **0.08** |  | **0.0/0.0/0.0** | | **0.0/0.0/0.0** | | **0.0/0.0/0.0** | **0.04** | | **0.01** | | **0.01** |  |
