# Supplementary material for: Nasopharyngeal tubes in pediatric anesthesia: Is the flow‐dependent pressure drop across the tube suitable for calculating oropharyngeal pressure?
Source: Paediatr Anaesth. 2021 May 6;31(7):809–19. doi: 10.1111/pan.14194 (PMC8252547; doi:10.1111/pan.14194)
Supplement: Supplementary file 4 — Appendix S1 [file PAN-31-809-s001.docx]

**Appendix S1**

**Experimental setups: Flow and** Δ**P measurements**

We chose to test three uncuffed endotracheal tubes (Rusch, Teleflex Medical, Ireland), Mansfield, MA, USA) measuring 3.5, 4.0 and 5.0 mm inner diameter, 3.5 mm for infants, 4.0 mm for young children at risk of obstructive apnoea and 5.0 mm for older children with neurological disabilities. Because K_1_ and K_2_ vary according to the tube curvature, we approximated the NPT curvature corresponding to the nasal intubation route, by passing the NPT in a larger, elbow-shaped conduit (Figure 1, left panel). In this first *in vitro* determination of ΔP_NPT_, the NPT end was left open to the atmosphere so that Pressure at the distal end (Pdist) equalled zero and ΔP equalled Paw. To prevent errors in measuring Pdist owing to accelerative pressure losses (Bernoulli effect) that could give a faulty measurement (negative pressure), we inserted the distal NPT end in an open tube.^9^ The Pdist measuring site was placed as far as possible (6 to 8 cm) away from the flow expansion and exit site and Pdist equalled zero. All positions were optimized in preliminary laboratory investigations. Pdist was considered as the reference pressure signal.

Paw was measured from the lateral wall of a pressure-measuring connecting tube joined to the NPT (Figure 1, left panel) and connected to a differential pressure transducer (SensorTechnics 144SM070D-PCB, SensorTechnics, Inc., Mansfield, CA, USA). Flow was measured by a pneumotachograph (4500 series, non-heated, 0–35 LPM; Hans Rudolph, Kansas City, MO, USA), interfaced to a differential pressure transducer (SensorTechnics 144LU01D-PCB, SensorTechnics, Inc., Mansfield, CA, USA) and interposed between an anaesthetic machine (Primus, Dräger, Lübeck, Germany) and the pressure measuring connecting tube. Pdist was measured from openings located in the walls of the tube in which the NPT was inserted. Flow and pressure signals were sampled using an oscilloscope (PicoScope 4824, Pico Technology, UK).

Because K_2_ and K_1_ also vary according to the gas type, ΔP _NPT_ was characterized using the gas compositions more commonly used in clinical practice, such as oxygen and sevoflurane in oxygen at 2% concentration (2%Sevo/O_2_), 4% (4%Sevo/O_2_), 6% (6%Sevo/O_2_), 8% (8%Sevo/O_2_), (Sevorane, Abbott Laboratories, UK) delivered by an anaesthetic machine at flow rates from 0 L/min, in 1 L/min steps, to 20 L/min in a time span of 200 seconds for each characterization. Gas was delivered with the generator proximal to the NPT to determine positive coefficients and distal to the NPT for negative coefficients ^1^. Unlike previous studies that characterised the pressure-flow relationship across the tube using an oscillatory flow ^2^, because we worked in an open system with reduced oscillations we characterized the pressure-flow relationship using a QSF as previously described ^3^.

With the Set-up B, we simulated the clinical scenario of differently aged-children undergoing NPT-assisted anaesthesia using 3.5, 4.0 or 5.0 inner diameter tubes, under two degrees of mouth opening (large leak, inner diameter 4.0 mm, and small leak, 2.5 mm). We simulated two respiratory conditions: spontaneous breathing under CPAP at 30 breaths per minute and 50 mL tidal volume and three delivered flow (5, 8, and 12 L/min), and apnoea condition assisted by IPPV with two peak inspiratory pressure levels (15 and 25 cmH_2_O), zero PEEP, inspiratory time 1 sec, 30 breaths/min, set flow 5 L/min. CPAP was delivered using a modified T-tube with an adjustable resistance connected to the anaesthetic machine (Primus, Dräger, Lübeck, Germany) for delivering oxygen and oxygen mixed with sevoflurane through the NPT, and thus modify pressure levels (the expiratory resistance was kept constant). We also simulated IPPV by setting the ventilator on the anaesthetic machine in the pressure control ventilation mode (PCV). To detect the pressure and flow signals needed to calculate oropharyngeal pressure, we inserted the distal NPT in an artificial pharynx connected either to a motor-driven lung-model ^4^ used as a pump to simulate sinusoidal breathing flow during CPAP or to a test lung (Quick lung junior, IngMar, Medical, 10 mL/cm H_2_O compliance and 5 cm H_2_O /L/s resistance) during IPPV (Figure 2, set-up B, left panel). The artificial pharynx had an opening that simulated an open mouth. All the experiments were repeated using oxygen, 2% sevo or 4% sevo in oxygen.

Paw and flow were measured as in set up A.

**References**

1. Windisch W, Buchholt A, Stahl CA, Sorichter S, Guttmann J. Flow-dependent resistance of nasal masks used for non-invasive positive pressure ventilation. Respirology 2006 Jul;11(4):471-6.
2. Spaeth J, Steinmann D, Kaltofen H, Guttmann J, Schumann S. The pressure drop across the endotracheal tube in mechanically ventilated pediatric patients. Paediatr Anaesth. 2015; 25: 413-420.
3. Wright PE, Marini JJ, Bernard GR. In vitro versus in vivo comparison of endotracheal tube airflow resistance. Am Rev Respir Dis. 1989;140:10-6.
4. Montecchia F, Midulla F, Papoff P. A flow-leak correction algorithm for pneumotachographic work-of-breathing measurement during high-flow nasal cannula oxygen therapy. Med Eng Phys. 2018;54:32-43.
